# Impact of Comprehensive Health Resort Treatment on Insomnia and Quality of Life in Older Adults: A Pre-Experimental Study

**DOI:** 10.3390/healthcare14142085

**Published:** 2026-07-12

**Authors:** Grzegorz Onik, Magdalena Dąbrowska-Galas, Galina Mratskova, Dariusz Górka

**Affiliations:** 1 Department of Physical Medicine, Faculty of Health Sciences in Katowice, Medical University of Silesia in Katowice, 40-055 Katowice, Poland; 2 Department of Kinesitherapy and Special Methods, Faculty of Health Sciences in Katowice, Medical University of Silesia in Katowice, 40-055 Katowice, Poland; mdabrowska-galas@sum.edu.pl; 3 Department of Physical and Rehabilitation Medicine and Sport, Faculty of Medicine, Trakia University, 6000 Stara Zagora, Bulgaria; galina.mratskova@trakia-uni.bg; 4 Department of Sports Medicine and Physiology of Physical Effort, Faculty of Health Sciences in Katowice, Medical University of Silesia in Katowice, 40-055 Katowice, Poland; dgorka@sum.edu.pl

**Keywords:** older adults, insomnia, quality of life, health resort treatment, balneotherapy

## Abstract

**Introduction:** Sleep disorders are common in older adults, and quality of life is often diminished due to gradual declines in physical and mental abilities. Among the treatment strategies routinely applied in older adults, comprehensive health resort treatment is considered a promising option because of its multifaceted nature and low risk of adverse effects. **Objective:** The primary objective of this study was to evaluate the impact of comprehensive health resort treatment on insomnia and quality of life in older adults. The secondary objective was to examine whether treatment-induced changes in these parameters were associated with participants’ age. **Material and Methods:** The study group consisted of 83 participants (14 men and 69 women) with a mean age of 71.94 ± 6.00 years who underwent a 21-day comprehensive, individually tailored health resort treatment for degenerative joint disease. Insomnia was assessed using the Athens Insomnia Scale. Health-related quality of life was evaluated with the European Quality of Life Five Dimensions—Five Levels (EQ-5D-5L) instrument. Assessments were conducted twice: upon admission to and discharge from the sanatorium. **Results:** The mean Athens Insomnia Scale (AIS) score at baseline was 6.97 ± 6.21 across all participants. Following treatment, the AIS score decreased to 4.78 ± 4.95 (*p* < 0.0001). The baseline mean EQ-5D-5L index was 0.956 ± 0.064. After health resort treatment, the mean EQ-5D-5L index increased to 0.969 ± 0.042 (95% CI: 0.006 to 0.022; *p* = 0.001). Age was not associated with the magnitude of changes in the AIS score, EQ-5D-5L index or EQ-5D-5L VAS in the studied population. **Conclusions:** Comprehensive health resort treatment was associated with improvements in insomnia severity and quality of life in older adults, with similar changes observed across age groups. These findings suggest that comprehensive health resort treatment may be a beneficial component of geriatric health care.

## 1. Introduction

The global population of older adults is expanding rapidly [1]. Older adults commonly experience sleep problems, particularly insomnia, which can significantly deteriorate their quality of life [2,3]. According to the diagnostic criteria of the International Classification of Sleep Disorders, insomnia is defined as difficulty initiating or maintaining sleep, accompanied by daytime impairment. These sleep problems must occur at least three times per week and persist for at least three months [4]. The International Statistical Classification of Diseases and Related Health Problems, 10th Revision (ICD-10), requires symptoms to be present for at least one month and not be better explained by another psychiatric or sleep–wake disorder [2].

Approximately 12% to 57% of older adults meet the criteria for insomnia disorder, and sleep difficulties are more prevalent in females than in males across all age groups [5,6,7,8]. Sleep patterns change across the lifespan. Total sleep time declines with age, decreasing from 10 to 14 h per night in the pediatric age range to approximately 6.5 to 8 h per night in young adulthood, and further declining to about 5 to 7 h per night in older adults, where it plateaus around the age of 60 [8]. Reduced sleep duration and consequently decreased slow-wave (NREM3) and REM sleep are associated with impairments in memory, learning, and cognitive recovery [9]. Older adults typically experience earlier morning awakenings, earlier onset of sleepiness, and more frequent nocturnal awakenings after sleep onset. As a result, they are more likely to take daytime naps and ultimately experience a decline in sleep efficiency, which negatively affects mood, anxiety symptoms, social functioning, and overall quality of life [10,11,12].

Several interventions have been shown to improve sleep in older adults. Cognitive-behavioral therapy for insomnia (CBT-I) is recommended as the first-line treatment for insomnia in adults. When CBT-I is ineffective, pharmacological treatments may be considered [11]. Previous studies suggest that increased levels of structured social engagement and physical activity have beneficial effects on sleep and overall functioning in older adults [13,14,15]. However, the impact of physical therapy methods and comprehensive health resort treatment on insomnia in older adults has not yet been established. Therefore, the primary objective of this study was to evaluate the impact of comprehensive health resort treatment on insomnia and quality of life in older adults. The secondary objective was to examine whether treatment-induced changes in these parameters were associated with participants’ age. We hypothesized that health resort treatment would be associated with improvements in insomnia severity and quality of life in older adults. Furthermore, we hypothesized that treatment efficacy might differ according to participants’ age.

## 2. Materials and Methods

### 2.1. Study Group

**Figure 1:**
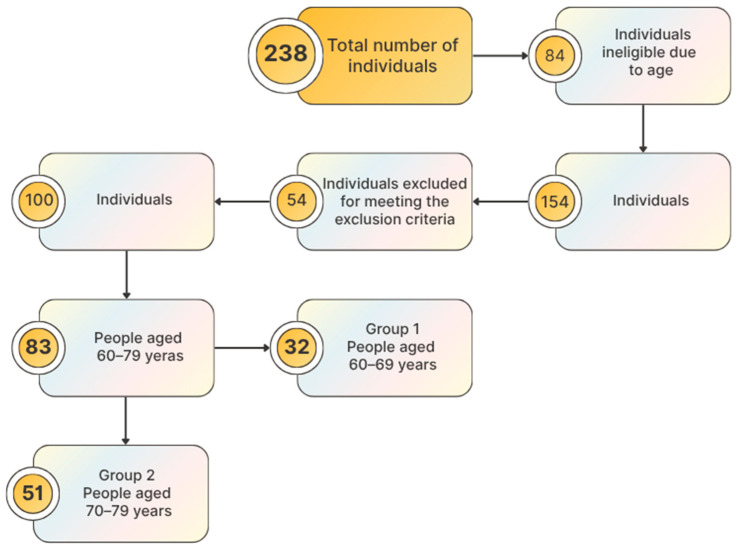
Flow diagram of participant selection.

Between February and June 2025, 238 individuals underwent comprehensive health resort treatment. Of these, 154 met the age inclusion criterion, and 54 were excluded because they fulfilled the exclusion criteria. Ultimately, the study included 100 participants aged 40–79 years (21 men and 79 women). For subsequent analyses, only individuals aged 60–79 years were included. No a priori sample size calculation was performed (Figure 1).

The final study group consisted of 83 participants (14 men and 69 women) with degenerative joint disease and a mean age of 71.94 ± 6.00 years. The mean body mass index (BMI) was 28.96 ± 4.46 kg/m^2^ (range: 21.63–42.86). The mean systolic blood pressure was 141.66 ± 7.05 mmHg (range: 112–155), whereas the mean diastolic blood pressure was 77.46 ± 6.06 mmHg (range: 60–90). At admission, the mean heart rate was 73.27 ± 8.34 bpm (range: 46–88).

Participants were divided into two age groups: individuals aged 60–69 years (Group 1, *n* = 32) and those aged 70–79 years (Group 2, *n* = 51). Men accounted for 25% of participants in Group 1 and 12% in Group 2, with no significant difference between the groups (*p* = 0.12). Hypertension was the most prevalent comorbidity, affecting 55.42% of the study population. Hypertension was significantly more common in Group 2 than in Group 1 (χ^2^ = 4.62; *p* = 0.03). Group characteristics are presented in Table 1.

Participants underwent a 21-day comprehensive health resort treatment program. The treatment protocol was individually tailored based on a medical examination conducted within 24 h of admission. Participants were reassessed after 7 days of treatment to monitor potential adverse effects or paradoxical reactions and underwent a final examination at discharge. In accordance with national spa treatment regulations, all participants received therapy 6 days per week, with at least three treatment procedures per day. Each treatment day included at least one balneological modality, such as brine or peloid therapy, in addition to other physiotherapeutic interventions. Participants also received education on healthy lifestyle habits, avoidance of harmful behaviors, and recommendations regarding physical activity and diet. All participants attended exercise sessions supervised by a physiotherapist.

Peloid therapy was the most frequently administered balneological modality, with 96% of participants receiving it. It was applied at 40 °C for 15 min. A statistically significant difference between the age groups was observed only for brine, which was administered to 88% of participants aged 70–79 years compared with 64% of those aged 60–69 years. Brine was administered at 38–40 °C for 15 min. A detailed overview of the frequency of balneological and physiotherapeutic modalities is presented in Table 2.

### 2.2. Inclusion and Exclusion Criteria

Individuals aged 40–79 years who underwent complete, comprehensive health resort treatment were eligible for the study. Exclusion criteria included: cardiovascular diseases (heart failure NYHA > II, atrial fibrillation, history of myocardial infarction, coronary artery bypass grafting, percutaneous coronary intervention with stent placement, or cardiac pacemaker implantation); neurological disorders (Parkinson’s disease, multiple sclerosis, paresis or spasticity, history of stroke, meningitis, intellectual disability); respiratory system conditions (chronic obstructive pulmonary disease, pneumoconiosis); amputations; and cancer. Multiple exclusion criteria were used to reduce the risk of confounding.

### 2.3. Methods of Evaluation

The severity of insomnia was assessed using the Athens Insomnia Scale (AIS), which was developed on the basis of ICD-10 diagnostic criteria for insomnia. This validated self-report questionnaire comprises eight items assessing difficulty initiating sleep, awakenings during the night, early-morning awakening, total sleep duration, overall sleep quality, next-day well-being, next-day mental and physical functioning, and daytime sleepiness. The total AIS score ranges from 0 to 24 points [16,17,18]. A cut-off score of ≥6 has been shown to provide high diagnostic accuracy for the identification of insomnia, with a sensitivity of 93% and a specificity of 85% [19]. A validated Polish version of the AIS has also been developed [20].

The European Quality of Life Five Dimensions–Five Levels (EQ-5D-5L) instrument was used to assess health-related quality of life. This instrument assesses five dimensions of health: mobility, self-care, usual activities, pain/discomfort, and anxiety/depression [21]. Self-rated health status was assessed using the EQ-5D-5L Visual Analogue Scale (EQ-5D-5L VAS), a vertical scale ranging from 0 to 100, with higher scores indicating better health status [22]. Population reference values for Poland were reported by Golicki and Niewada in 2017 [23]. The EQ-5D-5L index was calculated according to the algorithm proposed by Golicki et al. [24]. The use of the EQ-5D-5L was registered with the EuroQol Research Foundation under registration number 70086.

### 2.4. Data Collection

Measurements were performed twice: at baseline and after completion of the 21-day comprehensive health resort treatment program. The assessments were carried out during the baseline and post-treatment medical examinations. A physician interviewed participants regarding their symptoms, medical conditions, insomnia severity, and health-related quality of life. Questionnaires were administered by the physician rather than self-completed by participants to ensure the reliability of the collected data, as some individuals might have experienced difficulties accurately interpreting questionnaire items. Participants agreed to the treatment protocol and evaluation. The Bioethical Committee of the Medical University of Silesia in Katowice determined that formal ethical approval was not required due to the non-experimental nature of the study (decision no. KNW/NWN/0052/KB/41/25, issued on 4 February 2025).

### 2.5. Outcome Variables

The primary outcomes were treatment-related changes in insomnia severity and health-related quality of life, assessed using the Athens Insomnia Scale (AIS), the EQ-5D-5L index, and the EQ-5D-5L VAS. Secondary outcomes included age-related differences in treatment response and the identification of factors associated with treatment effectiveness. Explanatory variables comprised age group, age as a continuous variable, sex, body mass index (BMI), comorbidities, systolic blood pressure, diastolic blood pressure, heart rate, and the therapeutic interventions administered during health resort treatment. These variables were included in multivariable regression models to evaluate their associations with treatment outcomes.

### 2.6. Statistical Analysis

Statistical analyses were performed using Stata 19 (StataCorp LLC, College Station, TX, USA). Continuous variables were summarized as mean ± standard deviation (SD), whereas categorical and ordinal variables were presented as frequencies and percentages. Change scores (Δ) were calculated as pre-treatment minus post-treatment values (pre−post). For the Athens Insomnia Scale (AIS), lower scores indicate lower insomnia severity; therefore, positive Δ values reflect improvement. In contrast, for the EQ-5D-5L index and EQ-5D-5L VAS, higher scores indicate better health status. Consequently, negative Δ values (pre−post) represent improvement.

Normality of continuous variables was assessed using the Shapiro–Wilk test. For normally distributed variables, within-group comparisons were performed using paired Student’s *t*-tests, whereas between-group comparisons were conducted using independent-samples Student’s *t*-tests. For variables that did not meet the assumption of normality, the Wilcoxon signed-rank test was used for within-group comparisons and the Wilcoxon rank-sum test for between-group comparisons. Comparisons between age groups were performed using unadjusted statistical tests without adjustment for potential covariates. Effect sizes were quantified using Cohen’s d and reported with 95% confidence intervals (CIs). Cohen’s d values of 0.2, 0.5, and 0.8 were interpreted as representing small, medium, and large effects, respectively. Effect estimates were reported with corresponding 95% CIs.

Multiple linear regression analyses were performed to examine whether participants’ age was associated with changes in outcome measures following treatment. The dependent variables were change scores (Δ) for the Athens Insomnia Scale, EQ-5D-5L index, and EQ-5D-5L VAS. Age was included as the independent variable of interest. All models were adjusted for sex, body mass index (BMI), comorbidities, systolic blood pressure, diastolic blood pressure, heart rate, and age group. Regression coefficients (β), R^2^ values, F statistics, and *p*-values were reported. Multivariable logistic regression analyses were performed to evaluate the association between therapeutic interventions and the likelihood of clinical improvement. Clinical improvement was defined as a binary outcome (1 = improvement, 0 = no improvement) derived from change scores (Δ) according to predefined, scale-specific criteria for the AIS, EQ-5D-5L index, and EQ-5D-5L VAS. Participants received different combinations of therapeutic interventions during treatment. Treatment modalities administered to fewer than 10% of participants in both age groups were excluded from the regression models to ensure model stability and reduce the risk of sparse-data bias. This approach was applied to minimize overfitting and improve the reliability of parameter estimates. Exercise therapy was excluded from the regression analyses because it was administered to all participants and therefore showed no variability.

Statistical significance was defined as *p* < 0.05.

## 3. Results

**Figure 2:**
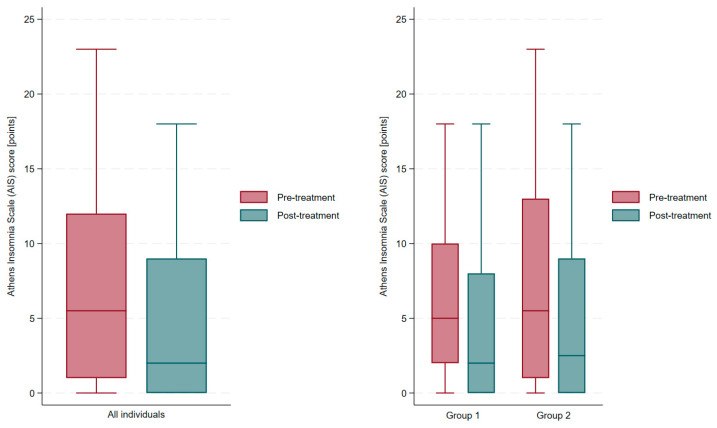
Comparison of insomnia severity, assessed using the Athens Insomnia Scale (AIS), before and after health resort treatment.

At baseline, insomnia defined according to the Athens Insomnia Scale (AIS) was identified in 50.60% of participants. In Group 1, 50.00% of individuals met the criteria for insomnia, compared with 50.98% in Group 2, with no statistically significant difference between the groups (χ^2^ = 0.008; *p* = 0.93). The mean baseline AIS score for the entire cohort was 6.73 ± 6.21 points. Following comprehensive health resort treatment, this value decreased by approximately 30%, from 6.73 ± 6.21 to 4.72 ± 4.89 points. This reduction was statistically significant (mean difference: 2.01 ± 3.05; t = 5.97; 95% CI: 1.35 to 2.68; *p* < 0.0001). In Group 1, the AIS score decreased by approximately 38% (mean difference: 2.38 ± 3.00; t = 4.47; 95% CI: 1.29 to 3.46; *p* = 0.0001). In participants aged 70–79 years (Group 2), the AIS score decreased by approximately 25% following treatment (mean difference: 1.78 ± 3.09; t = 4.07; 95% CI: 0.90 to 2.66; *p* = 0.0002) (Figure 2).

**Figure 3:**
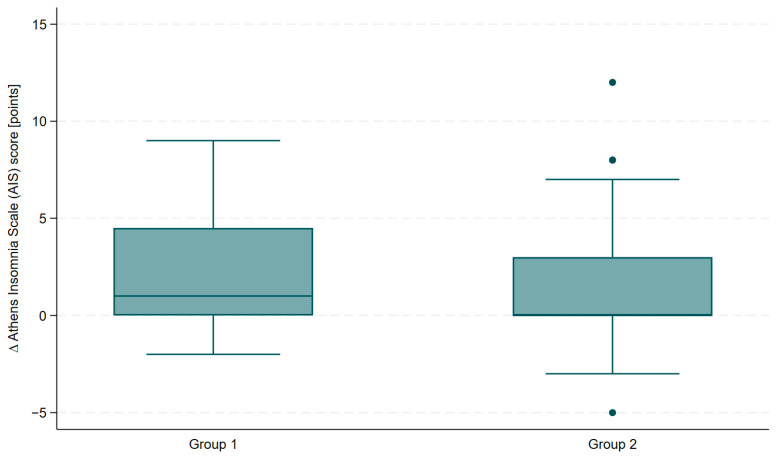
Comparison of changes in Athens Insomnia Scale (∆AIS) scores between groups.

The mean ΔAIS score was 2.38 ± 3.00 points in participants aged 60–69 years and 1.78 ± 3.09 points in those aged 70–79 years. Although the mean ΔAIS score was numerically higher in the younger group, no statistically significant difference between groups was observed (mean difference: 0.60 points; t = 0.86; 95% CI: −0.78 to 1.97; *p* = 0.39) (Figure 3). Consistent with this finding, effect size analysis showed a small between-group effect (Cohen’s d = 0.19; 95% CI: −0.25 to 0.64).

In both Groups 1 and 2, most participants reported no problems with mobility, self-care, or usual activities at baseline. Furthermore, approximately 90% of participants in both groups reported no problems in the anxiety/depression domain of the EQ-5D-5L. Although no statistically significant differences between groups were observed in the pain/discomfort domain at baseline, 46.67% of participants in Group 1 and 63.83% of those in Group 2 reported pain/discomfort. Comprehensive health resort treatment resulted in a significant improvement in the pain/discomfort domain in both groups. During the post-treatment assessment, no participants in Group 1 reported problems with self-care, whereas slight self-care problems were reported by 17.02% of participants in Group 2 (z = −2.33; *p* = 0.02). The distribution of responses across individual EQ-5D-5L domains is presented in Table 3.

**Figure 4:**
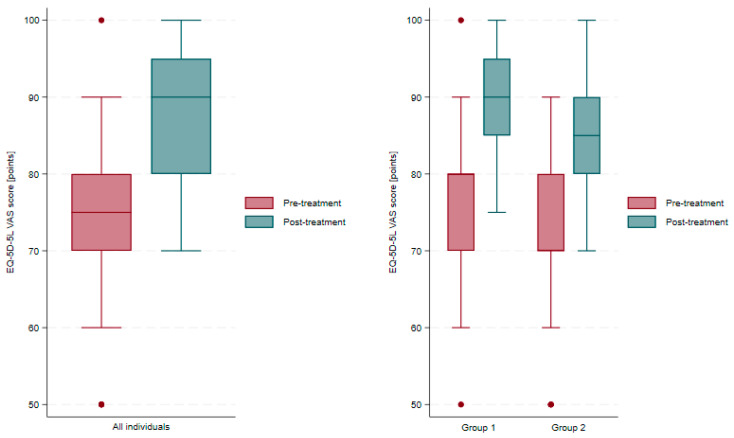
Comparison of EQ-5D-5L VAS scores at admission and discharge.

**Figure 5:**
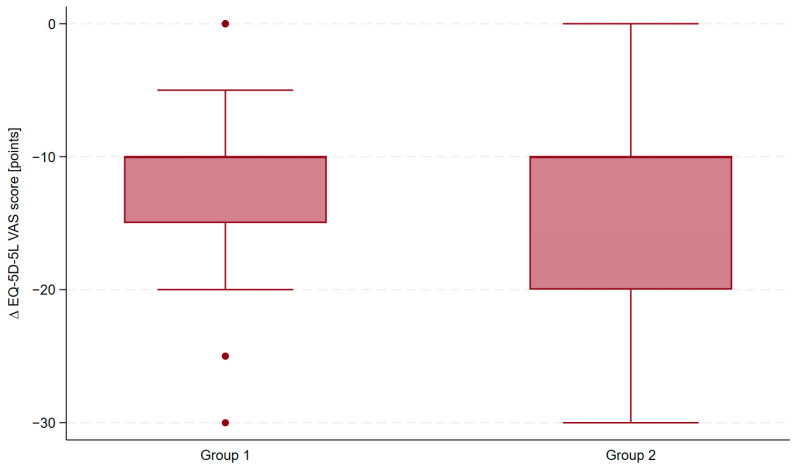
Comparison of changes in EQ-5D-5L VAS (∆EQ-5D-5L VAS).

In all participants, the mean pre-treatment EQ-5D-5L VAS score was 74.40 ± 10.31 points. At baseline, Group 1 had approximately 6% higher EQ-5D-5L VAS scores compared with Group 2 (mean difference: 5.05; t = 2.22; 95% CI: 0.53 to 9.57; *p* = 0.03). After the stay in the sanatorium, the EQ-5D-5L VAS score increased in all participants by approximately 15% (mean difference: –12.77 ± 6.31; t = −18.45; 95% CI: −14.15 to –11.39; *p* < 0.0001). In Group 1, the EQ-5D-5L VAS score improved by approximately 15% (mean difference: −12.19 ± 7.06; t = −9.76; 95% CI: −14.73 to −9.64; *p* < 0.0001), as did the score in Group 2 (mean difference: −13.18 ± 5.83; t = −16.10; 95% CI: −14.77 to −11.50; *p* < 0.0001). Similarly to the baseline measurement, participants in Group 1 had higher EQ-5D-5L VAS scores compared with those in Group 2 (mean difference: 4.09; t = 2.32; 95% CI: 0.58 to 7.62; *p* = 0.03). Figure 4 presents the EQ-5D-5L VAS scores at baseline and at the final evaluation, both for the overall study population and stratified by group. Although EQ-5D-5L VAS scores differed significantly between groups at discharge, the between-group difference in change scores (ΔEQ-5D-5L VAS) was not significant (mean difference: 0.95; t = 0.67; 95% CI: −1.89 to 3.79; *p* = 0.51). Figure 5 illustrates the ΔEQ-5D-5L VAS scores in both groups.

**Figure 6:**
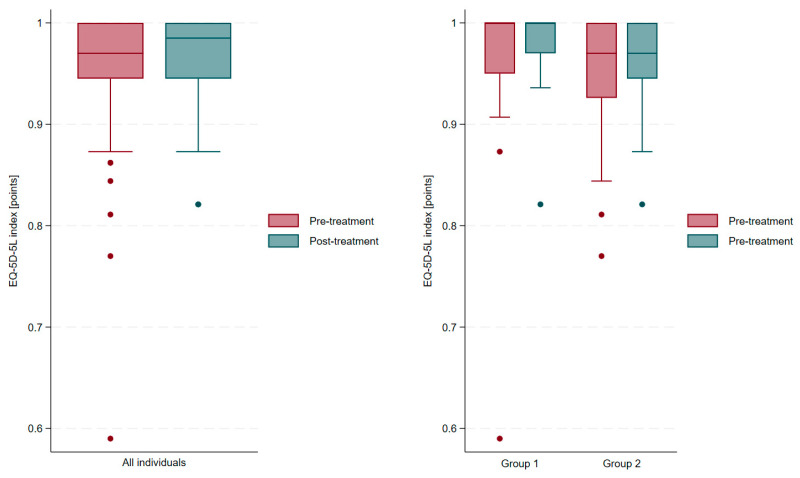
Comparison of EQ-5D-5L index at admission and discharge.

**Figure 7:**
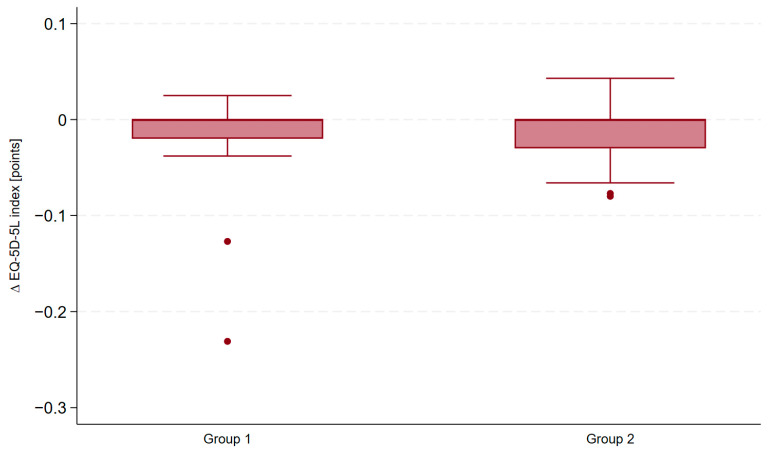
Comparison of changes in EQ-5D-5L index (∆EQ-5D-5L index).

In all participants, the mean baseline EQ-5D-5L index was 0.956 ± 0.064. After the health resort treatment, the mean EQ-5D-5L index increased to 0.969 ± 0.042 (mean difference: −0.014; t = –3.38; 95% CI: −0.022 to −0.006; *p* = 0.001). Comparison of baseline EQ-5D-5L index between the groups did not reveal significant differences (mean difference: 0.009; t = 0.60; 95% CI: −0.021 to 0.039; *p* = 0.55). Similarly, after the intervention, the groups did not differ in EQ-5D-5L index (mean difference: 0.014; t = 1.38; 95% CI: −0.005 to 0.033; *p* = 0.17). In individuals aged 60–69 years, the EQ-5D-5L index increased by approximately 2% after the treatment, which was not statistically significant (mean difference: −0.016 ± 0.049; t = −1.80; 95% CI: −0.035 to 0.002; *p* = 0.08). In participants aged 70–79 years (Group 2), the EQ-5D-5L index improved by approximately 1% (mean difference: −0.012 ± 0.024; t = −3.46; 95% CI: −0.019 to –0.005; *p* = 0.001) (Figure 6). In Group 1, the mean ∆EQ-5D-5L index was −0.016 ± 0.049, whereas in Group 2 it was −0.012 ± 0.024 (mean difference: −0.004; t = −0.52; 95% CI: −0.021 to 0.012; *p* = 0.59). Effect sizes for the difference in the ∆EQ-5D-5L index between age groups were very small. Cohen’s d −0.12 (95% CI: −0.59 to 0.34) (Figure 7).

**Figure 8:**
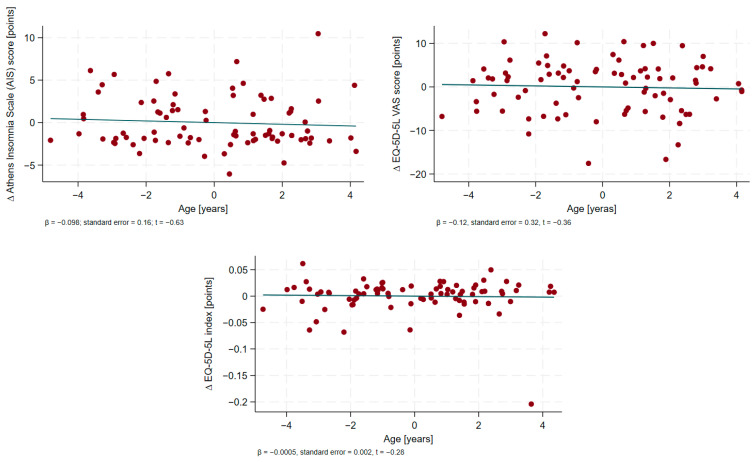
Adjusted associations between participants’ age and changes in AIS, EQ-5D-5L index, and EQ-5D-5L VAS scores.

Multivariate regression analysis revealed that age was not associated with the magnitude of changes in the Athens Insomnia Scale (AIS) (F(8,73) = 0.48; R^2^ = 0.05; *p* = 0.87), the EQ-5D-5L index (F(8,73) = 1.26; R^2^ = 0.13; *p* = 0.28), or the EQ-5D-5L VAS (F(8,74) = 0.66; R^2^ = 0.07; *p* = 0.72), after adjustment for sex, body mass index (BMI), comorbidities, systolic blood pressure, diastolic blood pressure, heart rate, and age group. Figure 8 presents the results of multivariable regression analyses examining the association between participants’ age and changes in AIS, EQ-5D-5L index, and EQ-5D-5L VAS scores after adjustment for confounders.

**Figure 9:**
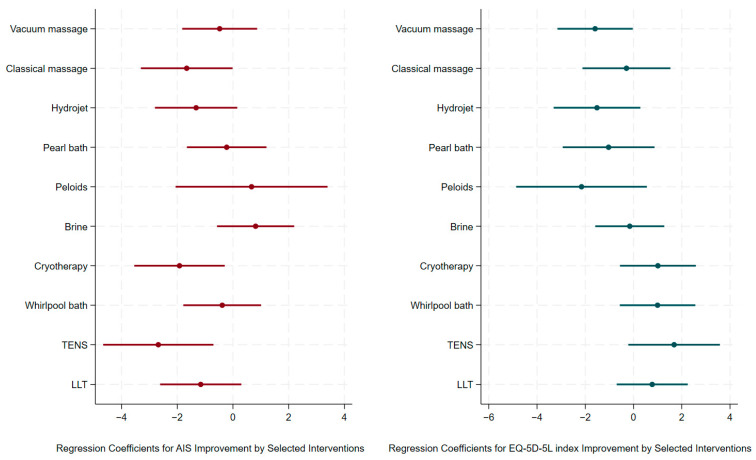
Coefficient plots of therapeutic interventions associated with improvements in Athens Insomnia Scale (AIS) and EQ-5D-5L index.

In the multivariable logistic regression model assessing the association between the application of particular modalities and improvement in AIS, the overall model was statistically significant (LR χ^2^ = 23.36; pseudo R^2^ = 0.20; *p* = 0.02). Classical massage (OR = 0.19; 95% CI: 0.037 to 0.99; *p* = 0.048) and transcutaneous electrical nerve stimulation (TENS) (OR = 0.06; 95% CI: 0.009 to 0.50; *p* = 0.008) were significantly associated with lower odds of improvement in AIS compared with no treatment. No statistically significant associations were observed for the remaining therapeutic modalities or age group (OR = 0.55; 95% CI: 0.17 to 1.81; *p* = 0.33). In the second multivariable logistic regression model (LR χ^2^ = 25.31; pseudo R^2^ = 0.25; *p* = 0.008), examining the association between specific interventions and improvement in EQ-5D-5L index, vacuum massage was significantly associated with lower odds of improvement in EQ-5D-5L index (OR = 0.20; 95% CI: 0.043 to 0.97; *p* = 0.046). Transcutaneous electrical nerve stimulation (TENS) showed a positive association with improvement (OR = 5.38), although this result did not reach statistical significance (95% CI: 0.81 to 35.89; *p* = 0.08). No statistically significant associations were observed for the remaining therapeutic modalities or age group (OR = 0.78; 95% CI: 0.22 to 2.79; *p* = 0.70). Figure 9 presents coefficient plots illustrating the associations between selected therapeutic interventions and improvement in AIS and EQ-5D-5L index. No significant associations between applied modalities, age group, and improvement in EQ-5D-5L VAS were observed, as the multivariable logistic regression model was not statistically significant (LR χ^2^ = 4.89; pseudo R^2^ = 0.19; *p* = 0.67).

## 4. Discussion

The principal finding of this study is that comprehensive health resort treatment was associated with improvements in insomnia symptoms among older adults, irrespective of age. In addition, multimodal health resort interventions are associated with beneficial effects on health-related quality of life, particularly through the alleviation of pain and discomfort and the enhancement of overall perceived health status.

Upon baseline assessment, individuals qualified for health resort treatment were found to have insomnia based on the AIS cut-off score. This finding is consistent with previous reports demonstrating that insomnia is highly prevalent among older adults [2,25,26]. To date, numerous factors have been identified as being associated with insomnia. Among these, female sex has been recognized as an important determinant [25]. In our study, 83% of participants were women, which may have contributed to the prevalence of insomnia in the study population. Moreover, participants enrolled in the study had comorbidities that are also associated with insomnia, including diabetes, hypertension, and cardiovascular disease [26]. Therefore, these conditions may have contributed to the occurrence of insomnia in the studied population. Although it was not the primary aim of the study, we found that individuals aged 70–79 years exhibited more severe insomnia symptoms than their younger counterparts. This finding is consistent with reports indicating that insomnia is more common among older individuals and that its prevalence increases with age [2,26].

After health resort treatment, insomnia severity was lower in the studied individuals. Although the reduction in the Athens Insomnia Scale score was greater in younger participants, no statistically significant between-group difference in effect size (Cohen’s d). Several hypotheses may be proposed to explain these findings. According to Polish health resort treatment regulations, treatment is conducted in designated areas that must meet specific criteria, including the enforcement of night-time quiet hours to ensure an uninterrupted therapeutic environment [27]. The presence of night-time quiet hours may be a contributing contextual factor associated with reduced insomnia symptoms, as irregular sleep schedules have been linked to impaired sleep quality [26]. Furthermore, health resort hospitals and sanatoria are located in zone “A”, which is required to consist of at least 65% green areas [27]. During their stay, individuals not only undergo medical procedures but also engage in walking in green spaces [28]. Previous studies suggest that walking is associated with better sleep outcomes and that exposure to green landscapes may additionally improve sleep-related parameters [29,30]. These contextual and behavioural factors may have contributed to the observed improvements in insomnia in the studied population.

During the sanatorium stay, all participants attended exercise sessions as well as physical and balneological treatments. Previous reports indicate that physical activity and exercise have positive effects on sleep quality, particularly by reducing sleep onset latency and improving sleep efficiency and continuity [31,32,33]. Several mechanisms may underlie exercise-mediated improvements in insomnia, including neurotransmitter synthesis and action, activation of the hypothalamic–pituitary–adrenal axis, anti-inflammatory effects, thermoregulatory responses, as well as stress reduction and mood improvement [34,35]. Balneotherapy, including procedures such as mineral water baths and mud therapy, has also been identified as a factor associated with improved sleep outcomes [36,37]. Castelli et al. [38] suggested that balneotherapy-related thermoregulatory responses may underlie sleep improvement, as body temperature is regulated according to the circadian rhythm. Given the hypothesis that inflammatory processes, particularly those mediated by interleukin-6, contribute to sleep disorders, the application of balneological treatments may further explain sleep-related improvements [39] through reductions in pro-inflammatory cytokines and increases in anti-inflammatory mediators [28]. Moreover, high temperature applied during balneological procedures may contribute to activation of the hypothalamic–pituitary–adrenal axis [40]. Additionally, balneological treatments have been shown to improve autonomic nervous system regulation [41], which may further contribute to sleep improvement, as insomnia is associated with altered autonomic regulation [42].

In the present study, most participants underwent brine baths and mud therapy. Uemura et al. [43] demonstrated that bathing in sodium chloride-rich water is associated with improved sleep, potentially due to enhanced heat conservation and prolonged tissue warming after immersion, as sodium chloride remains on the skin. Brine treatments have also been reported to increase heat dissipation and reduce core body temperature, which may be associated with subsequent improvements in sleep-related outcomes. Mud therapy has been shown to exert multiple biological effects related to the thermal properties of peloids; thus, it may influence thermoregulation and be associated with sleep-related improvements [44]. Moreover, peloids have been reported to reduce serotonin levels and exert anti-inflammatory effects, which may ultimately contribute to improved sleep, considering the bidirectional relationship between sleep and inflammation [39,45]. Although brine treatments were more frequently administered in group II, this was not associated with greater improvements in sleep outcomes. Overall, the observed improvement in insomnia may reflect the combined effects of multiple therapeutic modalities, as health resort treatment represents a complex, multimodal intervention [46].

At the pre-treatment assessment, younger participants reported higher overall health scores, as assessed by the EQ-5D-5L VAS scale. Age-related functional and cognitive limitations may substantially influence self-perceived health among participants assigned to Group 2 [47,48]. However, no statistically significant differences between the groups were observed at baseline in the remaining EQ-5D-5L domains. This finding may reflect legal requirements stipulating adequate self-care ability and independent mobility as criteria for referral to health resort treatment [49]. Although comprehensive sanatorium treatment was associated with improvements in the EQ-5D-5L index, the magnitude of the effect was comparable between the age groups. Our results are consistent with available reports demonstrating improved quality of life following balneological treatment in health resort settings across different medical conditions [50,51,52,53]. Nevertheless, it is important to note that participants had relatively high pre-treatment values of the EQ-5D-5L index, and although the improvement was statistically significant, its clinical relevance remains uncertain. However, considering that a 21-day stay in a health resort was associated with an increase in overall health status, as assessed by the EQ-5D-5L VAS, the observed changes in the EQ-5D-5L index may indicate a potential improvement in health-related quality of life.

In all participants, comprehensive health resort treatment was associated with a reduction in pain and discomfort, without favouring either age group, as changes in pain and discomfort (Δ) did not differ between groups, and Cohen’s d indicated a very small effect size. Overall, physical medicine modalities, balneological factors, and therapeutic exercises administered during the sanatorium stay may contribute to reductions in pain, which could partly account for the observed findings [28,46,51,54]. Although pain reduction may involve multiple mechanisms, the frequent use of brine and mud therapy in the study group suggests that thermal effects may play a role in this process. Heat may increase the pain threshold of nerve endings, inhibit nociception through gate control mechanisms, and stimulate the release of β-endorphins [51], potentially contributing to pain relief. Additionally, indirect effects such as enhanced blood flow, reduced muscle tone, and decreased inflammation may further support analgesic outcomes [28,46,51]. Despite these favourable changes in pain and discomfort, pain is a well-established factor associated with reduced quality of life [55,56]; however, in the present study, no significant association was observed between changes in pain or discomfort (Δ) and changes in the EQ-5D-5L index (ΔEQ-5D-5L). Similarly, no association was found between changes in pain or discomfort and changes in the AIS score (ΔAIS), although previous studies have reported a relationship between pain and sleep quality [57,58]. We also did not observe a significant association between participants’ age and changes in the Athens Insomnia Scale, EQ-5D-5L index, or the EQ-5D-5L VAS. These findings suggest that age was not significantly associated with the magnitude of improvement in sleep quality or quality of life following health resort treatment.

Considering the heterogeneous treatment courses among the study participants, a multivariable logistic regression model was applied to assess the association between specific therapeutic modalities and clinical outcomes. Classical massage and TENS were associated with lower odds of insomnia improvement. These findings are inconsistent with those reported by Ntoumas [59] and Akpinar [60], who suggested that massage may effectively improve sleep quality. Similarly, TENS has also been considered a modality potentially beneficial for sleep outcomes [61,62]. TENS was additionally associated with lower odds of improvement in the EQ-5D-5L index. The lower odds of outcome improvement may be related to the multimodal nature of health resort treatment; therefore, the observed associations may reflect the effects of co-administered therapeutic modalities rather than the isolated effect of a single intervention.

The present study has several limitations. Due to the application of numerous exclusion criteria, the generalizability of the findings may be limited. Nevertheless, we excluded medical conditions that are known to influence sleep and quality of life. The study included a relatively small sample size. Future studies should recruit larger samples to improve the reliability and generalizability of the findings. Moreover, the study did not include a control group (i.e., individuals not receiving health resort treatment). Therefore, the observed changes cannot be attributed exclusively to the intervention, as they may have been influenced by non-specific factors such as regression to the mean, repeated measurement effects, or expectancy effects. Future studies including a control group are warranted to allow a more rigorous assessment of the causal effects of health resort treatment on health outcomes. Finally, the study did not include a follow-up assessment. Future research should incorporate follow-up measurements to evaluate the durability of the observed treatment effects over time. Participants in the present study underwent individually tailored treatment courses, which may affect the reproducibility and interpretation of specific treatment effects; however, a unified treatment protocol should be implemented in future studies to strengthen the study design. Pain intensity was not assessed using dedicated tools such as the VAS or NRS scales. Future studies should include systematic pain assessments to better establish the relationships between outcomes and pain. Moreover, sleep disorder assessment did not account for potential sociopsychological factors, socioeconomic status, medication use (e.g., painkillers or hypnotics), alcohol and tobacco consumption, physical activity, or daytime napping, which may have influenced baseline measurements. Future studies should include these socioeconomic and lifestyle variables, including health-related behaviours. Considering legal regulations requiring self-care ability and independent mobility as criteria for referral to health resort treatment, the study findings may not be fully generalizable to frailer geriatric populations. Due to high baseline scores on the EQ-5D-5L, a ceiling effect may have been present. In contrast, a potential floor effect should be considered for insomnia severity, as participants exhibited relatively low AIS scores at admission.

## 5. Conclusions

Comprehensive health resort treatment was associated with reductions in insomnia severity and improvements in quality of life in older adults, with observed benefits across age groups. Given these findings, this form of comprehensive health resort treatment may be considered a beneficial intervention for older populations. However, further controlled studies are required to confirm these findings and establish causal effectiveness.

## Figures and Tables

**Table 1:** Characteristics of the participants.

Anthropometric Variable	Group 1 (*n* = 32)			Group 2 (*n* = 51)			*t*	*p*
	Min	Max	Mean ± SD	Min	Max	Mean ± SD		
Age [years]	60	69	65.13 ± 2.83	71	79	76.22 ± 2.38	−19.22	*p* < 0.0001
Height [m]	1.55	1.83	1.67 ± 0.08	1.46	1.88	1.63 ± 0.08	2.39	0.02
Weight [kg]	57	115	81.31 ± 16.83	54	112	76.73 ± 13.36	1.38	0.17
BMI [kg/m^2^]	21.63	42.86	29.10 ± 5.42	21.83	40.16	28.86 ± 3.80	0.23	0.82
SBP [mmHg]	112	155	141.00 ± 8.48	126	153	142.08 ± 6.04	−0.68	0.50
DBP [mmHg]	62	87	77.53 ± 6.51	60	90	77.41 ± 5.82	0.09	0.93
HR [bpm]	52	88	74.78 ± 7.63	46	88	72.31 ± 8.60	1.32	0.19
Comorbidity			Group 1 (*n* = 32)		Group 2 (*n* = 51)		χ^2^	*p*
Osteoporosis			9%		12%		0.12	0.73
Hypothyroidism			19%		27%		0.81	0.37
Hyperthyroidism			0%		6%		1.95	0.16
Hypertension			41%		65%		4.62	0.03
Depression			0%		6%		1.95	0.16
Mitral regurgitation			3%		4%		0.04	0.85
Ischemic heart disease			9%		12%		0.12	0.73
Varicose veins			13%		18%		0.39	0.53
Diabetes mellitus			13%		18%		0.39	0.53
Gastroesophageal reflux disease			0%		4%		1.29	0.26
Hyperuricemia			13%		8%		0.49	0.48
Gout			0%		2%		0.64	0.43
Benign prostatic hyperplasia			3%		4%		0.04	0.85
Peripheral artery disease			0%		2%		0.64	0.43

Legend: Continuous variables are presented as mean ± standard deviation (SD); categorical variables are presented as percentages; *t*—*t*-statistic; χ^2^—chi-square statistic; *p*—*p*-value.

**Table 2:** Frequency of balneological factors applied in the study group.

Procedure	All (*N* = 83)	Group 1 (*n* = 32)	Group 2 (*n* = 51)	χ^2^	*p*
Vacuum massage	54%	66%	47%	2.73	0.10
Classical massage	13%	19%	10%	1.37	0.24
HydroJet	22%	31%	16%	2.80	0.09
Pearl bath	23%	25%	22%	0.13	0.72
Peloids	96%	97%	96%	0.04	0.85
Brine	78%	63%	88%	7.67	0.006
Cryotherapy	15%	9%	18%	1.09	0.30
Whirlpool bath	20%	19%	22%	0.09	0.76
Infrared light	6%	0%	10%	3.34	0.07
Magnetledtherapy	5%	6%	4%	0.23	0.63
TENS	23%	9%	14%	0.35	0.55
Low-level laser therapy	22%	13%	28%	2.59	0.11
Ultrasounds	6%	6%	6%	0.005	0.94
Iontophoresis	6%	6%	6%	0.005	0.94
LED therapy	2%	0%	4%	1.29	0.26

Legend: Variables are presented as percentages; χ^2^—chi-square statistic; *p*—*p*-value.

**Table 3:** EQ-5D-5L domains across patient groups at admission and discharge.

Group 1	Mobility		Self-Care		Usual Activities		Pain/Discomfort		Anxiety/Depression	
	Before	After	Before	After	Before	After	Before	After	Before	After
No problems	80%	82.76%	93.33%	100%	93.33%	82.98%	53.33%	55.17%	90.00%	100.00%
Slight problems	13.33%	10.34%	6.67%	0%	6.67%	14.89%	23.33%	44.83%	10%	0%
Moderate problems	3.33%	3.45%	0%	0%	0%	2.13	20.00%	0%	0%	0%
Severe problems	3.33%	3.45%	0%	0%	0%	0%	3.33%	0%	0%	0%
Extreme problems/unable to do	0%	0%	0%	0%	0%	0%	0%	0%	0%	0%
*z*	0.00		1.00		1.00		2.83		1.41	
*p* ^1^	1.00		0.32		0.32		0.005		0.16	
Group 2	Mobility		Self-care		Usual activities		Pain/discomfort		Anxiety/depression	
	Before	After	Before	After	Before	After	Before	After	Before	After
No problems	59.57%	65.96%	82.98%	82.98%	82.98%	87.23%	36.17%	53.19%	90.00%	100.00%
Slight problems	25.53%	25.53%	14.89%	17.02%	14.89%	12.77%	46.81%	44.68%	10.00%	0%
Moderate problems	12.77%	6.38%	2.13%	0%	2.13%	0%	17.02%	2.13%	0%	0%
Severe problems	2.13%	2.13%	0%	0%	0%	0%	0%	0%	0%	0%
Extreme problems/unable to do	0%	0%	0%	0%	0%	0%	0%	0%	0%	0%
*z*	1.90		0.58		1.34		3.87		0.00	
*p* ^1^	0.06		0.56		0.18		0.0001		1.00	
Group 1 vs. Group 2	Mobility		Self-care		Usual activities		Pain/discomfort		Anxiety/depression	
	Before	After	Before	After	Before	After	Before	After	Before	After
*z*	−1.82	−1.47	−1.33	−2.33	−1.33	−1.36	−0.77	−0.25	−0.09	−1.81
*p* ^2^	0.07	0.14	0.19	0.02	0.19	0.18	0.44	0.81	0.93	0.07

Legend: Variables are presented as percentages; *z*—Wilcoxon test statistic; *p*^1^—*p*-value for intragroup comparisons before vs. after treatment; *p*^2^—*p*-value for intergroup comparisons between Group 1 and Group 2.

## Data Availability

The data presented in this study are available from the corresponding author upon reasonable request due to privacy and ethical restrictions, as they contain potentially identifiable information.
